# An Exploratory Review of the Potential of Lytic Proteins and Bacteriophages for the Treatment of Tuberculosis

**DOI:** 10.3390/microorganisms12030570

**Published:** 2024-03-12

**Authors:** Sibongile Mtimka, Priyen Pillay, Lusisizwe Kwezi, Ofentse Jacob Pooe, Tsepo Lebiletsa Tsekoa

**Affiliations:** 1 Future Production: Chemicals Cluster, Council for Scientific and Industrial Research, Pretoria 0001, South Africa; 219094519@stu.ukzn.ac.za (S.M.); ppillay3@csir.co.za (P.P.); lkwezi@csir.co.za (L.K.); 2 Discipline of Biochemistry, School of Life Sciences, University of KwaZulu-Natal, Durban 4000, South Africa; pooeo@ukzn.ac.za

**Keywords:** phage-derived lytic proteins, *Mycobacterium tuberculosis*, antibiotic resistance

## Abstract

Tuberculosis (TB) is a highly prevalent infectious disease that causes more than 1.5 million deaths a year. More than 25% of TB deaths occur in Africa, and TB is South Africa’s leading cause of death, with about 89,000 people dying of it yearly. The emergence of multidrug-resistant TB (MDR-TB) poses a significant threat to health security and could reverse the positive gains already made in the fight against TB. Antibiotic treatments are available, but side effects and the alarming increase in the prevalence of drug-resistant strains of *Mycobacterium tuberculosis* (*Mtb*) will compromise the control of the spread and treatment of the disease. A promising option is to employ specialized enzymes encoded by bacteriophages, which destroy bacterial cell membranes and walls to treat tuberculosis. Phage therapy against bacteria is a known treatment that is now reemerging with lytic proteins. These proteins provide an alternative means to treat infectious diseases where conventional antibiotic regimens do not meet the requirements. This review explores and discusses the potential of lytic protein therapy as an antimicrobial strategy against *M. tuberculosis* and multidrug-resistant tuberculosis.

## 1. Introduction

The current antibiotic resistance crisis poses a significant global threat to public health. The emergence of antibiotic resistance is due to (i) overuse, (ii) inappropriate prescribing, (iii) extensive agricultural use, and (iv) the scarcity of new antibiotics/drugs. These factors have rendered many conventional antibiotics ineffective against infections. Multidrug resistance is caused by multiple biochemical factors and presents a serious challenge in fighting infectious diseases such as *Mycobacterium tuberculosis* (*Mtb*). The growing resistance to antibiotics is alarming, as it limits treatment options, prolongs illnesses, increases healthcare costs, and increases mortality rates. Infections that were once easily treatable are now life-threatening due to antibiotic resistance. If left unchecked, this trend could lead to a future in which common infections and routine medical procedures become dangerous and threaten lives. Furthermore, microbes can employ more than one mechanism, making it even more challenging to combat multidrug resistance.

Drug resistance develops in a number of ways, but the five major ones are: (i) mutation or enzymatic alteration of the bacterial target, (ii) inactivation of the antibiotic by enzymatic degradation and modifications, (iii) bypass by the bacterium of the inhibition mechanism, (iv) overexpression of the drug target, (v) inhibition or slowing down of drug penetration by certain groups of proteins and the cell wall, and (vi) efflux of the antibiotic efflux pumps [1,2]. The development of new antimicrobials, such as endolysins, is crucial to combating this crisis. Endolysins offer a promising solution by precisely targeting bacterial pathogens while potentially evading existing resistance mechanisms. Their ability to disrupt the walls of bacterial cells presents a unique approach that might help overcome the challenges posed by antibiotic resistance. Addressing this crisis requires immediate action in the research and development of alternative antimicrobial strategies, such as endolysins, to ensure continued effectiveness in combating tuberculosis infections and safeguarding public health. The field of phage lytic enzymes has been dramatically expanded, first for Gram-positive pathogens and later also for Gram-negative pathogens, through protein engineering [3]. Although a growing focus has been on lytic enzymes that target Gram-positive bacteria, there is a pressing need to address Gram-negative infections, where the outer membrane presents an obstacle. Currently, several strategies have been developed to break the barrier posed by the outer membrane of Gram-negative bacteria to natural lysins. Physical (i.e., high hydrostatic pressure) and chemical permeabilizers (i.e., EDTA and weak organic acids, usually citric acid) can efficiently permeabilize the outer membrane to enhance the antibacterial activity of lysins [4]. This review explores and discusses the potential of lytic proteins to act as antimicrobial agents against *M. tuberculosis* and multidrug-resistant TB.

## 2. Tuberculosis (TB), *Mycobacterium tuberculosis* (*Mtb*), and the Rise of Multidrug-Resistant TB

Initial cases of *M. tuberculosis* in humans were traced back to the consumption of infected meat or the intake of contaminated milk. *M. tuberculosis* has a complex cell envelope, which is a partially covalently linked composite of polysaccharides, peptidoglycans, and lipids, including a layer of mycolic acid, which enhances pathogenicity but also protects against antibiotics [5,6,7,8]. Tuberculosis (TB), caused by *Mycobacterium tuberculosis* (*Mtb*), is the leading cause of death in the world today, killing an estimated 1.8 people annually. TB manifests itself primarily as an endemic, chronic pulmonary disease that kills both young and old. This is compounded by drug-resistant tuberculosis, the most common and lethal airborne antimicrobial-resistant (AMR) disease worldwide today, responsible for an estimated 250,000 deaths per year. Resistance to *Mtb* can be attributed to random genetic mutations and not transposition or conjugation. Genetic factors of *Mtb* have little influence on how effective the treatment of TB can be, except for the presence of drug-resistant mutations [9]. Multidrug-resistant tuberculosis (MDR) is characterized by its resistance to both rifampin and isoniazid and is complex to treat. The emergence of multidrug-resistant TB continues to strain existing TB control measures.

Patients with multidrug-resistant tuberculosis (MDR-TB) require long and extensive multidrug therapy regimens that are expensive, toxic, and less effective than second-line medications. Furthermore, second-line medication for MDR-TB is in short supply, with just 52% of patients successfully treated globally. Second-line drug resistance has already disrupted treatment regimens for around 50% of MDR-TB patients around the world. In the extensive treatment of drug-resistant disease (XDR-TB), in the best case, only one in three patients is successfully cured. In the past 70 years, only two newly developed drugs for treating MDR-TB have reached the market, yet R&D funding for TB has been at its lowest since 2008. Patients exhibit prolonged pain and frequent chronic diseases, compounded by catastrophic economic hardship, related stigma, and unwarranted prejudice. [10,11]. Projections indicate that if the diagnosis and treatment of all MDR-TB in known active TB cases could be implemented using a new and effective second-line regimen, a 54% reduction in MDR-TB could be achieved in five years [12,13]. Although the treatment of MDR-TB or extensively drug-resistant TB (XDR-TB) has been a challenge, the global rate of success in treating MDR-TB is 55% [14,15]. Despite advances in the diagnosis of tuberculosis and drug susceptibility assessments, nearly 50% of patients still do not successfully complete their tuberculosis treatment. Various factors contribute to this effect, including adverse drug reactions, lengthy treatment regimens, treatment costs, stigma, and the assumption that one is cured or healed when symptoms resolve. The inability to culture the bacterium from patients’ sputum is also a factor [14,16,17,18]. The use of fluoroquinolones (levofloxacin or moxifloxacin), together with linezolid and bedaquiline is used as a baseline treatment for rifampicin-resistant or multidrug-resistant TB [19]. These treatments are ineffective in some cases; in others, the duration must be longer. Prolonged multi-agent treatments can contribute to widespread antimicrobial resistance, necessitating novel interventions to prevent and control the spread of this deadly condition [20,21]. The potential for toxicity from long regimens can also not be ignored.

## 3. Available Treatment Options for Tuberculosis

Clinical trials conducted by the UK Medical Research Council and the US Public Health Services between 1948 and 1986 showed that completing a six-month multidrug course led to the curing of drug-susceptible TB with minimal chances of relapse [9]. Inappropriate treatment results in relapse, usually within 12 months of the completion of the initial course [9,17]. Initially, rifampin, coupled with isoniazid, shortened therapy to nine months. The inclusion of pyrazinamide in the cocktail in the first two months further reduced treatment to six months [9,22]. The standard six-month treatment is broken down into two phases: the induction phase of two months consisting of at least isoniazid, rifampin, and pyrazinamide. This is followed by the consolidation phase in the remaining six months; this phase consists of at least isoniazid and rifampin [6,23]. The current standard induction phase regimen comprises rifampin, isoniazid, and pyrazinamide. Ethambutol is introduced into the phase to prevent unexpected resistance to any of the three medications and ceases to be administered upon verification of susceptibility to the treatment. Several reactions have been associated with the toxicity of TB drugs, including hepatotoxic effects, gastrointestinal disorders, allergic reactions, and arthralgias [24,25]. The World Health Organization (WHO) currently recommends that the duration of MDR-TB treatment be a minimum of 20 months; the induction phase should be six to eight months and should include four drugs together with pyrazinamide [6,9,26,27]. Drug susceptibility varies per patient, and the induction phase may be ineffective for some. Therefore, the consolidation phase is necessary to avoid relapse. The increase in drugs in the induction phase has yielded positive results in the treatment of MDR-TB. TB treatment should also be administered to patients with human immunodeficiency virus co-infection while on antiretroviral therapy (ART), and if TB was diagnosed before receiving ART, it is recommended to start ART weeks after initiating TB treatment [23,28,29]. The CD4+ T cell count of the patient determines the waiting period. Different combinations of drugs can be used in cases of resistance to one drug. For example, when resistance to isoniazid occurs without signs of resistance to rifampin, isoniazid is replaced by a later generation fluoroquinolone (levofloxacin or moxifloxacin) and a 6-month regimen containing rifampin, moxifloxacin, pyrazinamide, and ethambutol for two months, followed by rifapentine and moxifloxacin for four months [9]. Most TB therapeutic agents currently in development are classified as antibiotics. In May 2017, 51 antibiotics and 11 biologicals targeting priority pathogens, *M. tuberculosis* and *C. difficile*, were on the clinical pipeline [27].

## 4. Bacteriophages, Their Derivatives, and Their Therapeutic Potential

**Figure 1:**
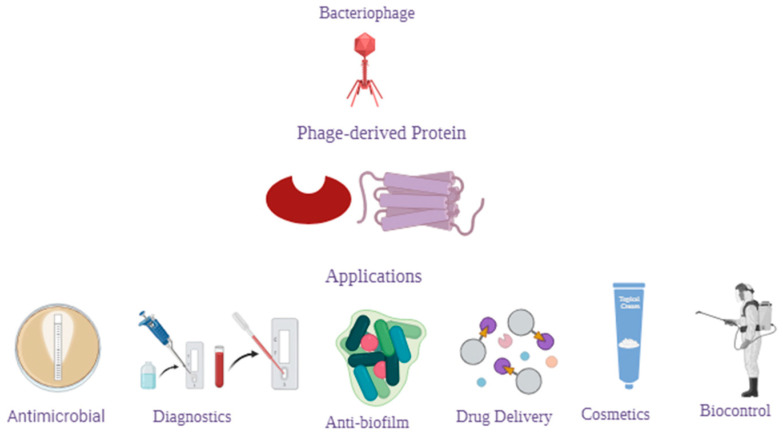
Schematic summary of potential applications of phage-derived proteins.

Bacteria-infecting viruses known as bacteriophages (phages) are found widely in nature. In their lytic life cycle, they kill their hosts. Over the years, this function has generated great interest in using them as antimicrobial agents to combat antibiotic resistance in bacteria [30,31]. Their application can be extended to numerous functions (Figure 1), including but not limited to: (i) detection of pathogens in the environment or food [32], (ii) disinfection of medical apparatuses and devices, (iii) prevention of the formation of bacterial biofilms on industrial surfaces [32,33,34], or (iv) vehicles for drug delivery [30,35,36].

Phage therapy involves treating bacterial infections with bacteriophages that infect and lyse bacteria to cure or prevent infectious diseases [37,38]. Using phages to treat tuberculosis is potentially advantageous, as they precisely target bacterial pathogens and do not adversely affect the host. Preclinical animal efficacy studies have demonstrated a success rate of up to 100% elimination of infections caused by MDR pathogens using phages [39]. However, a downside of using phages as antimicrobials is their ability to potentially reach a state of equilibrium with the target bacterium rather than eliminating it. This occurs because phages are obligate intracellular parasites whose replication and survival depend on host cell survival. It is also crucial to maintain adequate, but minimal, levels of phages when administering them as therapeutics. For example, in vitro studies have shown that exposing infection-causing bacteria to a high density of phages can lead to the elimination of administrative therapeutic phages, as exposure can lead to the selection of insensitive bacteria [40,41].

### 4.1. Phage-Derived Proteins

#### 4.1.1. Phage-Lytic Proteins

Bacteriophage peptidoglycan (PG) degrading enzymes are increasingly being investigated as antibacterial agents, with several products for topical use against Gram-positive infections already on the market [27,42]. They are classified into three groups: endolysins, holins, and virion-associated peptidoglycan hydrolases (VAPGHs). These phage-lytic proteins can infect Gram-positive and Gram-negative bacteria, making them good candidates for combating drug-resistant bacterial pathogens. This classification is based on the mode of action, which differs in some steps of the infection cycle [30,43]. The term endolysins was adopted after understanding the enzymes’ synthesis and sequestration within the cell cytoplasm, assembling phage particles [44]. Endolysins are proposed to be administered exogenously and represent an alternative form of treatment against antimicrobial resistance [45,46,47,48]. Given the promise of cell wall degrading enzymes in treating Gram-positive and Gram-negative infections, they have potential for treating *M. tuberculosis* that warrants further study [49,50]. Novel and practical options for TB treatment are critical, particularly for drug-resistant tuberculosis, as drug resistance continues to grow and cripple the global economy [51].

##### Endolysin-Peptidoglycan Degrading Enzymes

Endolysins, also known as peptidoglycan hydrolases, are a class of bacteriophage-encoded enzymes that degrade the cell wall of bacteria [5,48,52]. These enzymes can also cleave the bacterial peptidoglycan (PG) from within the bacterial cell, leading to the lysis of the bacteria [53]. Bacteriophage endolysins, also known as lysins, represent a novel group of antimicrobial agents that are increasingly being recognized as alternative options for preventing and treating bacterial infections. Lysins consist of a catalytic component responsible for breaking specific bonds in bacterial peptidoglycan and share a classification with hydrolases [54]. The binding domain allows each lysin to target a specific substrate in the bacterial cell wall (usually carbohydrate), offering some species specificity [55]. When applied exogenously to Gram-positive bacteria, native or recombinant lysins are capable of degrading the cell wall of susceptible bacteria and causing logarithmic cell lysis within seconds to minutes [55]. Lysins typically lack signal sequences, which means that in other cases, they may rely on holin to access the PG [33].

From a safety perspective, the high specificity of endolysins is among their most beneficial properties in food safety and medical applications. These enzymes specifically destroy a target pathogen without affecting the desired commensal microflora, conferring a significant advantage over many commonly used antibiotics or chemical preservatives [56,57]. A significant concern with systemic administration of lysins in humans or animals is the release of pro-inflammatory cellular debris associated with bacterial lysis, such as teichoic acids, lipoteichoic acids, and PG, which can cause serious adverse effects such as bacterial infection and failure of several organs [58]. Due to their protein-rich nature, endolysins are noncorrosive and biodegradable—this is considered an additional advantage [47,56,59] and it is not surprising that antibodies can be raised against these enzymes [52,56,60,61]. For example, when mice were immunized with the pneumococcal phage lysin Cpl, IgG was detected against phage lysin; however, there were no significant differences between immunized and naive mice regarding the reduction of bacterial numbers by the enzyme, suggesting that the antibodies produced were not able to inhibit lysin in vivo [62].

##### Holins-Cytoplasmic Membrane Degrading Enzymes

Holins are small transmembrane proteins that perforate the bacterial cytoplasmic membrane, and, in most cases [63,64], this process allows phage-encoded peptidoglycan hydrolases to act on the cell wall, resulting in host cell lysis and phage release [65]. Holin’s synergistic role thus involves inducing holes in the membrane, making the membrane permeable and susceptible to other lytic enzymes [63]. Their activity disrupts the membrane, thus enabling endolysin to pass through the inner membrane (IM) and target the peptidoglycan (PG). Holins exhibit significant diversity, often displaying unique amino acid sequences. Generally, they are compact proteins, usually containing fewer than 150 amino acids, and are characterized by at least one transmembrane domain (TMD). Additionally, holins typically possess a hydrophilic and highly charged C-terminal region [66]. Holins are categorized into three classes: I, II, and III, based on their mechanism of action deduced from the number of TMDs the holin possesses [63]. Class I holins are typically 90–125 amino acids in length, with three TMDs. In contrast, Class II holins are usually small, 65–85 amino acids in length, and have two TMDs. Class III holins typically have a single TMD. All holins share characteristics of primary sequences: a short N-terminal hydrophilic sequence, they potentially have dual starting motifs; a C-terminal domain rich in neutral pH-charged residues, and a connector sequence between the first two transmembrane domains with proline or glycine residues. Each class of holin plays a unique role in the success of bacterial cell lysis and in assisting endolysins to reach the PG [67].

##### VAPGH—Virion-Associated Peptidoglycan Hydrolases

VAPGHs act in the first phase of the lytic cycle, unlike endolysins, which act in the late phase of phage gene expression in the lytic cycle [30]. These phage-lytic proteins can target Gram-positive and Gram-negative bacteria [43], making them good candidates for combating drug-resistant bacterial pathogens. The primary purpose of VAPGHs is to assist in the release of newly formed phage progeny from the host bacterial cell by degrading the bacterial cell wall, which is composed of peptidoglycan. Their action is part of a coordinated process that involves other phage-encoded proteins such as holins and endolysins, and unlike endolysins, VAPGHs do not possess any cell-wall binding domain (CBD), but they have highly conserved domains [68].

#### 4.1.2. Therapeutic Potential of Phage-Lytic Proteins and Combination Therapy to Treat TB

**Figure 2:**
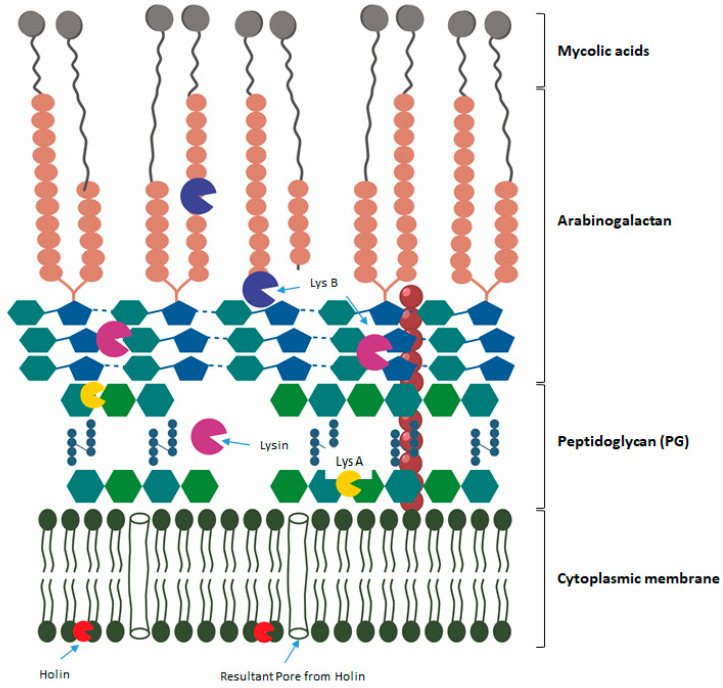
Structure of the *mycobacterium* cell envelope with a graphic elucidation of the biological/mode of action. LysA, which acts on the PG cell wall and hydrolyzes the glycoside bonds. LysB disrupts the bonds between mycolic acids and the arabinogalactan layer. Holin attaches to the cell wall, and the resultant pores are observed.

The susceptibility to a pathogen infection and the effectiveness of any antimicrobial therapeutic agent, including phages, are influenced by the individual’s immune status. Some mathematical models of the interactions of phages and bacteria propose the cooperation of the immune system for successful treatment using phage therapy [69,70]. Phage therapy for medical products has been withheld in some countries due to the demand for a high level of clinical evidence, regardless of the intrinsic efforts of academia, regulators, and biotech companies. One of the responses to high doses of phage treatment may be inflammation, triggered by the release of endotoxins. Due to the nature of phages, they stimulate an adaptive immune response. Their effects are typically caused by the toxicity of contaminants, e.g., lipopolysaccharides (LPS), from the preparation of phages [71]. For immunosuppressed patients, phage therapy may offer little benefit in targeting nosocomial infections. In particular, the amounts of LPS do not exceed those of antibiotic treatment [71,72]. Phage-based therapy for *M. tuberculosis*, even more so in humans, has revealed various outcomes within in vivo studies. The lack of study evidence may also be due in part to the intricate structure of the cell wall. As illustrated in Figure 2, the cell wall (CW) is made up of a thick layer of PG that is intrinsically linked to arabinogalactan that has been esterified with mycolic acids.

The ability of phage-lytic proteins to penetrate or degrade cell walls can facilitate treatment directly or through better drug delivery, which increases effectiveness and reduces the duration of therapy [7,66]. Lytic proteins are highly specific to the near-species or genus from which the phages are derived. Phage-lytic proteins isolated from phages infecting the genus *mycobacterium* show high specificity toward these species. For example, Holin Gp11, isolated from *Mycobacterium* phage D29, has been shown to cause cell lysis in both *Escherichia coli* and *M. smegmatis* when expressed at a low level and in the absence of endolysins [73]. Unique to mycobacteriophages is a biomembrane-targeting mycolylarabinogalactan esterase (LysB) to lyse mycobacteria [74,75,76]. These hold specific potential and can be used in cocktails with endolysins for therapeutic application in *M. smegmatis* or *M. tuberculosis*.

Phage-lytic proteins can be used in combination therapy to enhance the efficiency of the standard drug administered or reduce the dosage required for effective treatment. Lysogenic mycobacteriophages, for example, Mycobacteriophage Ms6, lyse the host with the cooperation of lysin and holin, ultimately releasing the new phage particles. Lin (2018) demonstrated bacterial lysis through the inducible production of cell wall hydrolyzing enzymes, mycobacteriophage lysins, and discovered that lysin induction produced lytic death in both replicating and non-replicating *Mtb* [77]. Genetically inactivating thioredoxin reductase was also shown to disrupt various growth-critical processes, including sulfur and DNA metabolism, and rapidly kill and lyse *Mtb* when combined with mycobacteriophage lysins [78]. It was also noted that to achieve effective lysis, both holin and endolysin from D29 and L5 phages were required. Lysin expression triggered lytic death in nonreplicating *Mtb*, indicating that dormant *Mtb* has to preserve the integrity of the cell wall and is therefore susceptible to lysin killing [78]. Payne (2010) focused on the mycobacteriophage LysA and the accessory lysis protein LysB, showing preliminary evidence of peptidoglycan hydrolytic ability, inducible cell lysis, and the inhibition of *M. smegmatis* by these proteins [76].

The development of lytic phages that meet regulatory requirements and meet clinical needs is crucial to combating bacterial and antibiotic-resistant pathogens. In the age of multidrug resistance, phage-lytic protein treatment can be seen as a complement or an alternative to antibiotics. Most phage species will employ two major protein classes during the lysis of the bacterial host. One is the transmembrane protein, holin, and the other is the peptidoglycan cell wall hydrolase, endolysin (lysin). These two proteins will work in tandem to trigger bacterial cell wall lysis [57,79]. Table 1 summarizes the phage-lytic enzymes with therapeutic potential. D29 was initially used in the diagnosis of TB [80] and later for therapeutic purposes. It has been an efficient candidate for TB therapy when delivered as an aerosol because it goes directly to the site of infection, thus reducing the burden of the bacterium. Phage-lytic proteins are known to be specific, and D29, Bxz2, L5, and TM4 have broad specificity within *Mycobacterium* spp. [81,82]. The phages display various mechanisms of action to achieve their goal, while little to none is known about their mechanism of action. Although little is known about its mechanism, the DS-6A phage is specific to *M. tuberculosis*, while both TM4 and D29 are specific to *M. tuberculosis* and *M. smegmatis*. A review by Azimi et al. (2019) details the mechanism of varying phage-lytic proteins and their effects, not only in *Mtb* but also in other *mycobacterium* strains of the BTCU-1, and reveals broad antimicrobial activity against *mycobacterium* strains [5,8]. The D29 phage-lytic protein is one of the few that is already commercially available for assays, not only for *M. tuberculosis*, but also for *M. bovis* and *M. avium* subspecies paratuberculosis [83,84,85]. A study conducted by Bajpai et al. (2018) on anti-*Mtb* phages started with several phages, revealing nine that have activity against *Mtb*. The morphology of these two lytic proteins resembles that of the Siphoviridae family. Mycobacteriophage lysis proteins could greatly benefit from supporting antibiotic treatment [7,57]. The availability of lytic proteins that show disruption of the outer membrane with no added outer membrane disruptors is a good starting point for addressing viable TB treatment. Disrupting the outer membrane eventually leads to bacterial death; this also opens combination therapy options, whereby lytic proteins can act as a delivery system for the current drug regime. This would reduce the duration of the treatment and also aid in combating drug-resistant TB. The biological agents presented in Table 1 in general demonstrate the potential of these lytic enzymes to act as ‘preventive prophylaxis’ to act as a complementary treatment option, as demonstrated by the modes of action displayed by BTCU-1_ORF7 and BTCU-1_ORF8 (Table 1). The suboptimal targeting of current treatment regimens is largely accounted for by the presence and protection conferred to the bacterium by the outer membrane, and therefore the inclusion of such molecules described in Table 1 could ameliorate this challenge.

## 5. Conclusions and Future Prospects

The rise in antibiotic-resistant bacteria and the slow development of new antibiotics are severely impacting the effectiveness of current treatments, especially against drug-resistant infections and diseases such as tuberculosis, now the top infectious disease killer. This critical situation underscores the urgent need for innovative antimicrobial solutions. Phage therapy, together with its derivatives, presents a potential alternative treatment that leverages the co-evolution of phages and their respective hosts and possesses the necessary tools to infect and kill specific types of bacteria, such as *Mtb*, circumventing drug-resistance and cytotoxicity. These proteins are capable of directly destroying the cell membrane and also aiding in drug delivery. This ability to assist in drug delivery makes them great candidates for combination therapy, whereby these phage-lytic proteins would shorten most treatment regimens and reduce the number of incomplete treatments and relapses, ultimately addressing the multidrug resistance problem. Future studies on the integration and lysis molecular mechanisms of mycobacteriophage and phage-lytic proteins could further facilitate the development of new antimicrobials and anti-TB drugs. The adoption and uptake of research efforts investigating these molecules could lead to more candidates reaching advanced clinical trials. Furthermore, the potential of endolysins (phage-lytic proteins) as antimicrobial agents also makes them good accompanying candidates to combat the looming growth of resistance against antibiotics. To combat the phenomenon of ‘superbug’ bacteria, the use of phage-derived products with added specificity and efficacy is an appealing solution for developing countries. In comparison to whole-phage particles and products, in the future, the use of synthetic phage particles and smaller engineered enzymes may be better at penetrating tissues, be non-infectious, less susceptible to bacterial resistance, and have a wider antibacterial spectrum. Other envisioned benefits are reduced biofilm formation and less immunogenicity. Through the study, development, and clinical testing of phage-lytic proteins, part of the answer can be realized in improving the current efforts of the pharmaceutical industry in combating the increase in antibiotic-resistant bacteria. In addition to the use of these molecules as therapeutic agents, their alternative utility value when formulated as antimicrobial cleaning agents can also be leveraged in many settings to further reduce the spread of infections.

## Figures and Tables

**Table 1:** Phages and phage-lytic enzymes in development for the treatment of TB.

Name of Lytic Enzyme (Synonym)	Model	Target Pathogen	Result Summary	Reference
BTCU-1_ORF7 and BTCU-1_ORF8	In vitro	*M. smegmatis*	Endolysin has been shown to effectively reduce TB infection, endolysins derived from the mycobacteriophage BTCU-1 have antimycobacterial activity	[5]
PK34	Mouse	*M. tuberculosis*	PK34 binds to the glycolipid and cleared TB in the mouse model and reduced level of proinflammatory cytokines	[86]
TM4	Mouse	*M. avium*, *M. tuberculosis*	Reduction of infection when lytic bacteriophage TM4 was delivered transiently kills both *M. tuberculosis* and *M. avium* residing within macrophages	[81]
LysB/Ms6	In vitro	*Mycobacterium* spp.	Growth inhibition with surfactants	[61]
LysB/Bxz2	In vitro	*Mycobacterium* spp.	Growth inhibition with surfactants	
LysA/BTCU-1	In vitro	*Mycobacterium* spp.	Intracellular killing of *M. smegatis*	
LysB/BTCU-1	In vitro	*Mycobacterium* spp.	Intracellular killing of *M. smegatis*	
Ms6		*M. smegmatis*	The Ms6 lysis cassette has five genes involved in disruption of mycobacterium outer membrane	[7]
Phage-D29-derived LysB	In vitro	*M. smegmatis*	Showed synergy with various anti-TB drugs against *M. smegmatis* cells	[87]
D29 phage	Mouse	*M. tuberculosis*	Significantly reduced the burden of *M. tuberculosis* at 24 h and 3 weeks post-infection compared to untreated mice.	[88]
D29 phage	Animal models	*M. tuberculosis*	Shows preventive measures against *M. tuberculosis* infection when administered in an inhalation formulation.	[89]
DS6A, TM4, D29, BTCU-1, SWU1 and Ms6	In vivo (guinea pigs)	*M. tuberculosis*, *M. ulcerans*, *M*. *avium*	Elimination of *M. tuberculosis*. Reduction of infection in a number of organs and lesions	[82,90]
T7, P4, PDRPv, Bo4, Bxz2	In vitro and in vivo	*M. smegmatisi*, *M. tuberculosis*, *M. bovis*	Reduction of infection in a number of organs and reduced lesions on some organs	[82,91]
